# 

**Published:** 1877-03

**Authors:** 


					﻿Vol. 24.__________MARCH, 1877.___________No. 3.
TWENTY-FOURTH YEAR.
HALL’S
Journal of Health.
PUBLISHED AT 137 EIGHTH STREET, NEW YORK,
Four Doors East of 750 Broadway.
E. H. GIBBS, M.D., Editor.
AGENTS FOB THE TBADE I
AMERICAN NEWS COMPANY, 121 NASSAU STREET.
NEW YORK NEWS COMPANY, 18 BEEKMAN STREET.
Terms, $ 1.50 a Year.
SINGLE NUMBER, 15 CENTS.
Kkmt & Co.. Printers, 11 Frankfort St.-, Naw York.
				

